# Lost in Translation: Simple Steps in Experimental Design of Neurorehabilitation-Based Research Interventions to Promote Motor Recovery Post-Stroke

**DOI:** 10.3389/fnhum.2021.644335

**Published:** 2021-04-20

**Authors:** Natalia Sánchez, Carolee J. Winstein

**Affiliations:** ^1^Division of Biokinesiology and Physical Therapy, University of Southern California, Los Angeles, CA, United States; ^2^Department of Neurology, Keck School of Medicine, University of Southern California, Los Angeles, CA, United States

**Keywords:** stroke, neurorehabilitation, recovery, compensation, impairment

## Abstract

Stroke continues to be a leading cause of disability. Basic neurorehabilitation research is necessary to inform the neuropathophysiology of impaired motor control, and to develop targeted interventions with potential to remediate disability post-stroke. Despite knowledge gained from basic research studies, the effectiveness of research-based interventions for reducing motor impairment has been no greater than standard of practice interventions. In this perspective, we offer suggestions for overcoming translational barriers integral to experimental design, to augment traditional protocols, and re-route the rehabilitation trajectory toward recovery and away from compensation. First, we suggest that researchers consider modifying task practice schedules to focus on key aspects of movement quality, while minimizing the appearance of compensatory behaviors. Second, we suggest that researchers supplement primary outcome measures with secondary measures that capture emerging maladaptive compensations at other segments or joints. Third, we offer suggestions about how to maximize participant engagement, self-direction, and motivation, by embedding the task into a meaningful context, a strategy more likely to enable goal-action coupling, associated with improved neuro-motor control and learning. Finally, we remind the reader that motor impairment post-stroke is a multidimensional problem that involves central and peripheral sensorimotor systems, likely influenced by chronicity of stroke. Thus, stroke chronicity should be given special consideration for both participant recruitment and subsequent data analyses. We hope that future research endeavors will consider these suggestions in the design of the next generation of intervention studies in neurorehabilitation, to improve translation of research advances to improved participation and quality of life for stroke survivors.

## Introduction

Stroke continues to be one of the leading causes of disability with around 800,000 new and recurring strokes occurring every year (Virani et al., 2020). Advances in early pharmacological interventions after stroke, such as the use of thrombolytic factors like tissue plasminogen activator (Lo et al., 2003), combined with longer life expectancy will markedly increase the number of survivors of stroke. After stroke, the primary functional qualifier for discharge eligibility from an in-patient rehabilitation facility is the ability to stand and walk independently (Bohannon et al., 1988, 1991; Olney and Richards, 1996; Fulk et al., 2017), and to achieve enough residual function to allow self-care and independence when performing basic activities of daily living (Duncan et al., 2005; Winstein et al., 2016). Even after intensive in-patient physical and occupational therapy, which may continue into outpatient settings, marked functional impairments remain in a majority of stroke survivors. Despite our unprecedented understanding of the neuropathophysiology of impaired motor control post-stroke and the recent development of technologies to quantify and assist motor function, research-based recovery-promoting interventions have not been any more successful than best-approach practices of physical and occupational therapy in mitigating the sensorimotor impairments that limit motor function in the long term (Pollock et al., 2014b; Corbetta et al., 2015; French et al., 2016; Langhorne et al., 2018; Hornby et al., 2020). After stroke, around 80% of people post-stroke show remaining walking impairment (Li et al., 2018; Mansfield et al., 2018; Virani et al., 2020), and 65% of stroke survivors do not incorporate their affected arm and hand into everyday activities (Mayo et al., 2002; Dobkin, 2005; Winstein et al., 2016). As a result, only 25% of survivors of stroke return to the level of activity and participation pre-stroke (Lai et al., 2002; Dobkin, 2005). Therefore, the problem of motor impairment post-stroke still needs effective solutions.

Stroke can result in sensory, cognitive, perceptual, emotional and language impairments (Winstein et al., 2016). This perspective focuses on sensorimotor impairments after stroke, but we acknowledge that a multidisciplinary team that allows targeting each of these multi-system impairments is an aspirational goal for the success of interventions in neurorehabilitation. Here, we offer our perspective as to why research in neurorehabilitation of movement has yet to change clinical practice of post-stroke therapy. To set up our perspective, we first define the terminology used here, which is consistent with the International Consensus panel: Stroke Recovery and Rehabilitation Roundtable (SRRR; Bernhardt et al., 2017). We refer to the survivor of stroke as participant (Krakauer and Carmichael, 2017), as our perspective is centered on research-based interventions. Rehabilitation is defined as “a process of active change by which a person who has become disabled acquires the knowledge and skills needed for optimum physical, psychological, and social function (Bernhardt et al., 2017),” with the term neurorehabilitation referring specifically to rehabilitation after nervous system injury. Recovery as defined by the SRRR, refers to a return to pre-stroke function (Bernhardt et al., 2017), and this process is driven by restitution, which requires neural reorganization and repair (Bernhardt et al., 2017; Krakauer and Carmichael, 2017). In agreement with the SRRR, recovery can occur in any domain of the International Classification of Functioning and Disability (ICF), which includes body function, body structure, activities and participation, and environmental factors (World Health Organization, 2002). Our focus here will be on recovery of body function, which likely impacts the other ICF domains. Compensation refers to the use of residual but not original capacity to substitute motor patterns that are nevertheless adaptive to task requirements (Levin et al., 2009; Bernhardt et al., 2017). Given these definitions, we offer a bold but realistic set of considerations grounded in a body of human and animal model research. We believe that if this set of considerations are adopted in future neurorehabilitation intervention studies, we as movement scientists can re-route the rehabilitation trajectory toward recovery-based behaviors and away from compensatory-based behaviors.

### Consideration 1: What Matters Is Not *if* Participants Can Accomplish a Task but *how* They Accomplish it

As stated in Krakauer and Carmichael (2017)’s exceptional book “Broken Movement,” most motor learning research in stroke has been performed under the assumption that movement practice will inherently lead to improvements in impairment (Krakauer, 2006; Krakauer and Carmichael, 2017). Under this assumption, researchers argue that task-specific practice is always associated with positive neuroplasticity such as cortical reorganization and reweighting of the neural mechanisms that mediate the control of movement (Kleim et al., 1998; Liepert et al., 2000). The idea that practiced movements will inherently lead to positive changes at the level of central nervous system is often referred to as a *bottom-up approach:* can repeated practice with movement patterns that resemble those before stroke lead to re-learning of these patterns? A potential limitation of the bottom-up approach is that it can promote compensatory strategies given that success is measured by task completion but not necessarily by *how* the task was accomplished. In contrast, a *top-down approach* (Gordon, 1987; Winstein et al., 2003; Horno et al., 2011), might be better suited to remediate sensorimotor impairment by shifting focus from the behavioral performance to the “*how,”* and the specific neurophysiological mechanisms (i.e., mechanism of action) that mediate movement.

The clear distinction between a bottom-up and a top-down approach might become blurry from a practical standpoint. For example, a given intervention can be designed from the perspective of a top-down approach, but it could easily morph into one that emerges from a bottom-up approach. An example of how a top-down approach might shift to a bottom-up approach is seen when the experimenter introduces external guidance as an assist during practice to achieve more neurotypical movements similar to those seen pre-stroke. External guidance includes systems such as visual biofeedback (Jonsdottir et al., 2007, 2010; Pollock et al., 2014b; Mansfield et al., 2018), mirror therapy (Hamdy et al., 1998), verbal feedback (Rendos et al., 2020), or assistive devices that physically guide the participant’s extremities (Husemann et al., 2007; Ellis et al., 2008; Bishop and Stein, 2013). Research has shown that external guidance is a top-down approach as it can engage the reward systems in the basal ganglia, working memory regions, and the mirror neuron system (Dobkin, 2004; Langhorne et al., 2011). Additionally, external guidance can engage the visuomotor network (Archer et al., 2018) and the cerebellum (Doya, 2000; Archer et al., 2018), and supplement impaired somatosensation post-stroke (Tate and Milner, 2010). The engagement of these neural pathways may lead to recovery-supportive cortical reorganization (Hamdy et al., 1998; Kleim et al., 1998; Liepert et al., 2000). However, an overreliance on these forms of external guidance can develop with time and thereby shift an intervention to a bottom-up approach, especially if the participant’s focus shifts from how the movement is performed to simply goal achievement, be it hitting a target, or completing one of hundreds of repetitions. Over-reliance on external guidance can hinder the development of an internal reference of correctness and thereby degrades learning that is measured when the feedback/guidance is no longer available such as for long-term retention and transfer (Winstein and Schmidt, 1990; Schmidt and Bjork, 1992). This might explain why despite multiple studies demonstrate immediate changes in task performance with external guidance during skill acquisition, few, if any studies have shown guidance interventions to be more beneficial than traditional therapy for durable learning effects (Woodford and Price, 2007; Pollock et al., 2014a). Focus on the “how” underlying task performance is also important for interventions based on frameworks such as the FITT (frequency, intensity, type, and time) model for exercise prescription (Lawrence and Kuypers, 1968). Special emphasis should be placed on whether the type of exercise is promoting practice of a maladaptive compensatory pattern that might hinder recovery in the long term. Therefore, we cannot assume that simply focusing on task repetition will inherently lead to restitution of optimal movement patterns. Attention to how the task is performed is still important.

A way to assess whether repeated practice can indeed lead to permanent changes in the neural control of movement is by including retention and transfer trials as part of training schedules, which allow participants to volitionally perform movements and encourage exploration and self-direction (Schmidt and Bjork, 1992). For example, a progressive reduction in guidance allows more engagement of the volitional problem-solving system (Winstein and Schmidt, 1990; Schmidt and Bjork, 1992), and supports participants’ autonomy to explore the task workspace in search of effective solutions to achieve the movement goal (Winstein and Kay, 2015). The evidence also suggests that when guidance is reduced, the learner becomes more engaged and motivated in the recovery process (Eng and Tang, 2007; Tretriluxana et al., 2013; Lewthwaite et al., 2018; Winstein et al., 2019): by reducing reliance on the guidance itself, the locus of control is shifted from external (e.g., focus on the feedback or external device) to internal (focus on the volitional control goal; Winstein et al., 1999; McNevin et al., 2000). This shift fosters better goal-action coupling, and is more likely to enable fundamental learning-based mechanisms such as the dopaminergic reward system (Wulf and Lewthwaite, 2016). Therefore, task repetition might effectively lead to changes in the neural control of movement, if the training protocol supports the participant’s autonomy in the learning process (e.g., decision making, problem-solving).

### Consideration 2: Stroke Impairment Is a Multifaceted Problem of Central and Peripheral Adaptations and the Influence of Each Depends on Stroke Chronicity

Stroke induced lesions to descending neural pathways leads to altered neuromotor control not only due to the lesion itself, but due to diaschisis (Carrera and Tononi, 2014), and reweighting of the multiple inputs to motor neurons to compensate for decreased corticospinal drive (Mansfield et al., 2018). These changes include overreliance on diffuse brainstem pathways for motor control (Lawrence and Kuypers, 1968; Davidson and Buford, 2004; Riddle et al., 2009; Zaaimi et al., 2012; Herbert et al., 2015; Owen et al., 2017; McPherson et al., ^®^) and increased activation of the contralesional motor cortex (McPherson et al., 2018). Changes in descending neural input not only lead to functional changes but also to structural changes at the muscle level. Immobilization immediately after the stroke event leads to muscle fiber atrophy, decreased muscle-force generating capacity in both extremities measured as early as 1 week post stroke (Harris et al., 2001; Newham and Hsiao, 2001), slow to fast muscle fiber type conversion (Lieber, 2000) and changes in muscle volume, measured at 3 weeks after the stroke event (Young et al., 2006). Recovery from immobilization ensues with decreased neural drive, which itself, leads to permanent muscle atrophy (Klein et al., 2010; Triandafilou and Kamper, 2012). These stroke-induced changes in muscle properties, combined with disuse atrophy due to overall decreased physical activity (Billinger et al., 2014; Danks et al., 2016) heighten the functional impairments in neuromotor control after stroke. Therefore, impairments post-stroke arise due to a vicious cycle of altered neural drive, altered muscle properties and altered function that feed into each other over the chronicity of the stroke.

It is evident then how the chronicity of the stroke will promote endurance of this impairment-promoting cycle: the more time from stroke onset, the more these neural, muscular and functional changes become ingrained. Only recently, have researchers reached a consensus regarding the language to refer to the timeline of stroke recovery (Bernhardt et al., 2017). The SRRR taskforce defines the acute phase of stroke recovery as 1–7 days post-stroke, and the subacute phase from 7 days to 3 months post-stroke, with most recovery occurring during these two phases (Dobkin, 2005; Krakauer et al., 2012). The SRRR defines the late subacute phase as 3 to 6 months and the chronic phase as more than 6 months post stroke onset. Based on this knowledge, the next logical conclusion is that research should aim to promote recovery and prolong the recovery window in the early stages post-stroke. This was in fact the goal of a recent randomized trial which aimed to assess the efficacy of a high intensity, high-dose, non-task oriented upper extremity neuro-animation therapy in patients up to 6 weeks after stroke onset, compared to high-dose occupational therapy and traditional occupational therapy (Ward et al., 2019). Results from this trial found that the outcomes of both the neuro-animation therapy and high dose occupational therapy were equivalent in terms of functional recovery of motor control, strength and reaching kinematics, and superior to traditional therapy. These findings are suggestive that high dose interventions in the acute and subacute phase can prolong and modify the recovery trajectory.

**FIGURE 1:**
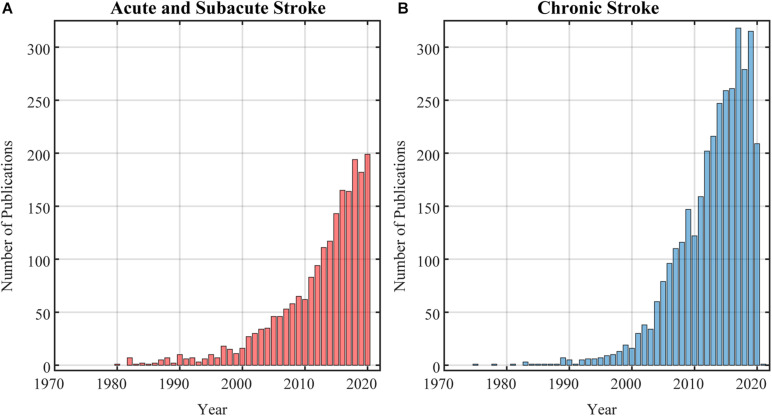
Results of a PubMed search on research studies in stroke neurorehabilitation as of December 1st, 2020. **(A)** Search returned 1789 publications in acute and sub-acute stroke. **(B)** Search returned 2968 publications in chronic stroke.

Impairment minimization is plausible (Ballester et al., 2019; Daly et al., 2019; Ward et al., 2019), but more complex in the chronic phase of stroke recovery, as the longer the time from stroke onset, the more practice survivors of stroke have had with maladaptive compensatory movement patterns. The majority of evidence for the effectiveness of non-pharmacological rehabilitation interventions has been generated in individuals in the chronic stage after stroke (Duncan et al., 2003). In a PubMed search of research in human movement rehabilitation after stroke, we found half as many published papers having enrolled individuals in the acute and sub-acute stages (Figure 1A), compared to the chronic stage of recovery (Figure 1B). From a practical standpoint, recruitment of stroke survivors in the chronic phase provides a larger population of individuals who are readily available for research studies: most stroke survivors in the chronic phase are not receiving regular therapy, nor are they part of the regular work-force (Cramer et al., 2017; Virani et al., 2020). In addition, research in the chronic stage is unlikely to be confounded by the natural recovery process (Krakauer et al., 2012). However, the potential efficacy of rehabilitation interventions might be underestimated since they are applied to a population where it may be more difficult to effectively promote recovery over compensation. We reason that the limited effectiveness of rehabilitation interventions delivered in the chronic phase of stroke might be because these interventions target a single mechanism of impairment to mitigate a multifaceted problem that involves both sensorimotor and muscular impairments overlaid upon a set of compensatory strategies that are well-learned and consolidated from repetitive use over the chronicity of the stroke. Thus, our recommendation is to consider stroke chronicity carefully during recruitment, and as a co-variate when analyzing response to an intervention, as the responsiveness of participants classified as chronic might vary significantly given the wide time window encompassed by the *chronic* definition, and wide range of sensorimotor symptoms engrained over that time.

### Consideration 3: Secondary Measures Are Needed to Track and Mitigate Emergence of Compensatory Behaviors During Interventions

Stroke-induced injury to the central nervous system impairs motor behaviors, leading to the emergence of compensatory strategies that use remaining sensorimotor elements to accomplish a task (Levin et al., 2009). Compensations can range widely from non-use to maladaptive use. For example, a stroke survivor may have the capacity to use their more affected hand to pick up a dime; however, they can accomplish this task faster and with less frustration using their trunk to lean closer to the dime, or even substituting for impaired hand function by use of their less affected hand (van Kordelar et al., 2012; Martinez et al., 2020). This compensatory strategy is known to provoke development of learned non-use of the paretic extremity even when capacity to use the paretic limb is sufficient (Taub et al., 2006). For locomotion, compensatory behaviors are characterized by maladaptive use of both lower extremities. An example is over-reliance on the less affected lower extremity for weight bearing (Olney and Richards, 1996) and forward propulsion (Roelker et al., 2019; Awad et al., 2020), leading to decreased used of the paretic extremity and associated reductions in bone mineral density (Worthen et al., 2005). Therefore, given the clear adverse long-term effects of these kinds of compensatory behaviors, targeted evidence-based interventions are needed to reverse or adapt maladaptive movement patterns in the chronic stages of recovery (Thompson and Wolpaw, 2014, 2019).

Tracking compensatory behaviors poses a major challenge for researchers. Compensatory behaviors will likely occur at joints or segments that are not the main focus of the research intervention study. Thus, researchers must ensure that experimental outcome measures can dissociate accurate task performance from compensation. Because multiple degrees of freedom (DOF) are enlisted with most functional behaviors, this means that there are multiple combinations of these DOF, that can be used to achieve the same endpoint. Since some of those combinations reveal compensatory solutions, we recommend that standard primary outcome measures are supplemented with secondary measures that capture how the movement is performed, such as joint kinematic or kinetic measures (Kwakkel et al., 2019; Martinez et al., 2020; Solnik et al., 2020). For example, joint kinematics can be used to quantify compensatory patterns that emerge with goal-directed paretic upper extremity movements, and joint kinetics can inform a compensatory over-reliance on the non-paretic lower extremity during walking. Inclusion of secondary measures to track compensatory behaviors might boost data complexity, but they are necessary to foster recovery-supportive movement solutions and to diminish compensatory-based movement solutions.

### Consideration 4: Patient-Driven Contextualization of Time-Intensive Interventions Can Lead to Changes in Impairment in the Chronic Phase of Stroke Recovery

Despite the generally accepted finding that recovery is unlikely if not already observed after the first 3 months post-stroke (Krakauer et al., 2012), recent studies have shown impairment mitigation in the chronic stage post-stroke. In a recent study, researchers administered 300 h of treatment (5 h/day, 5 days/week, over 12 weeks), which led to reduced impairment that was retained even at 3 months follow up (Daly et al., 2019). Another study (Ward et al., 2019) showed a marked decrease in upper limb motor impairment retained at 6 months follow up, after a multifaceted, intensive rehabilitation intervention of around 90 h (6 h/day, 5 days/week over 3 weeks). This intervention was aimed at re-education, task adaptation and building self-efficacy (intrinsic motivation) in the context of activities of daily living, which is known as a transfer package (Gauthier et al., 2008). Thus, high dose practice under optimal motivational and attentional focus conditions can increase motor performance and learning, and lead to neural changes that both decrease impairment and promote recovery (Wulf and Lewthwaite, 2016).

Under the current standard of care, it is unlikely for survivors of stroke to be able to receive the high dose of practice which has been shown to be more effective than traditional physical therapy practice (Lang et al., 2009; Pollock et al., 2014a). What remains to be determined is whether a transfer package that allows contextualization and development of self-management skills (Gauthier et al., 2008), along with optimal conditions of meaningful practice can invoke durable changes in recovery with a dosage that is consistent with the current standard of care. A recent study found that to maximize efficacy of task practice, practice should be given in relatively small bouts consisting of a high number of movements, with dosage that is personalized based on motor function distributed over months (Wang et al., 2020). Specifically, for those individuals with high motor function, practice during every day activities will continue to improve arm use, whereas for those with low motor function, there is need to develop a personalized transfer package to foster self-management (Wang et al., 2020). The importance of contextualized task practice highlights the need to establish outcomes that are meaningful to the participant and at the same time engage intrinsic motivation through autonomy support (agency) and attentional focus. In our own experience, participant’s goals include “not limping,” “having my gait look the same as everyone else’s,” “keeping my arm down as I walk,” “keeping my hand from automatically closing,” “being able to have the handwriting I used to have before the stroke,” and “changing my granddaughter’s diaper.” Note that a simple contextualization can drive motivation if the participant sees how the task can positively impact their life outside of the research lab, yet these person-centered outcomes are rarely if ever included in research protocols (Winstein, 2018). To further support our argument, recent studies have shown that motivation can mediate long-lasting neural plasticity: for example, operant conditioning protocols use a reward-based approach to downregulate plantarflexor muscle H-reflex through brain and spinal cord plasticity (Wolpaw et al., 1986; Thompson and Wolpaw, 2014; Chen et al., 2017), which can restore pre-injury reflex excitability and invoke positive changes in walking function (Thompson et al., 2013; Thompson and Wolpaw, 2019). Therefore, the contextualization process establishes meaningful goals linked to the research task being used to promote recovery through fundamental learning-based processes, supported by brain and spinal cord plasticity (Tsay and Winstein, 2020).

## Summary

Here, we offer a set of considerations for which there is evidence of recovery-based neural plasticity, to complement current experimental approaches. First, we advocate for a shift in focus from mere movement outcome/completion to a focus on capturing how the movement is performed (e.g., attention to the quality of movement) and coupled with permission to explore the workspace (i.e., self-practice) in search of effective solutions to achieving the movement goal (i.e., autonomy support). This permission to explore, make and correct errors is possible when training schedules allow participant exploration via a reduction in guidance and augmented feedback. Second, given the myriad of changes that occur over the chronicity of the stroke, researchers should control for stroke chronicity when assessing efficacy of a given intervention. To ensure that interventions are not promoting compensation, researchers should include secondary outcome measures that reveal how the movement was performed across linked segments and joints (e.g., kinematics). This scrutiny will aid in the identification and demotion maladaptive compensatory strategies and promote more restorative movement patterns. Finally, we suggest that researchers frame experimental protocols in a context that is meaningful to each participant; doing so will increase engagement, and improve learning through intrinsic motivation and reward which will drive recovery-based changes in neural function. These simple considerations can be implemented while not compromising the scientific rigor of research based intervention studies, and our hope is that in so doing, they can be used to re-route the rehabilitation trajectory toward recovery-based behaviors and away from compensatory-based behaviors.

## Data Availability Statement

The original contributions presented in the study are included in the article/Supplementary Material, further inquiries can be directed to the corresponding author/s.

## Author Contributions

NS and CW: conceptualization, writing – original draft preparation, and writing – review and editing. NS: funding acquisition. Both authors contributed to the article and approved the submitted version.

## Conflict of Interest

The authors declare that the research was conducted in the absence of any commercial or financial relationships that could be construed as a potential conflict of interest.
