# Supplementary material for: Lost in Translation: Simple Steps in Experimental Design of Neurorehabilitation-Based Research Interventions to Promote Motor Recovery Post-Stroke
Source: Front Hum Neurosci. 2021 Apr 20;15:644335. doi: 10.3389/fnhum.2021.644335 (PMC8093777; doi:10.3389/fnhum.2021.644335)
Supplement: Supplementary file 1 [file Data_Sheet_1.docx]

**Supplementary Materials**

Below is the PubMed detailed search query used to generate Figure 1.

1. *Search strategy for rehabilitation research in acute and subacute stroke*

**Search: ((((stroke) AND (rehabilitation OR neurorehabilitation)) AND (acute OR subacute)) NOT (chronic)) AND (movement OR motor)**

((("stroke"[MeSH Terms] OR "stroke"[All Fields] OR "strokes"[All Fields] OR "stroke s"[All Fields]) AND ("rehabilitant"[All Fields] OR "rehabilitants"[All Fields] OR "rehabilitate"[All Fields] OR "rehabilitated"[All Fields] OR "rehabilitates"[All Fields] OR "rehabilitating"[All Fields] OR "rehabilitation"[MeSH Terms] OR "rehabilitation"[All Fields] OR "rehabilitations"[All Fields] OR "rehabilitative"[All Fields] OR "rehabilitation"[MeSH Subheading] OR "rehabilitation s"[All Fields] OR "rehabilitational"[All Fields] OR "rehabilitator"[All Fields] OR "rehabilitators"[All Fields] OR ("neurological rehabilitation"[MeSH Terms] OR ("neurological"[All Fields] AND "rehabilitation"[All Fields]) OR "neurological rehabilitation"[All Fields] OR "neurorehabilitation"[All Fields] OR "neurorehabilitative"[All Fields])) AND ("acute"[All Fields] OR "acutely"[All Fields] OR "acutes"[All Fields] OR ("subacute"[All Fields] OR "subacutely"[All Fields]))) NOT ("chronic"[All Fields] OR "chronical"[All Fields] OR "chronically"[All Fields] OR "chronicities"[All Fields] OR "chronicity"[All Fields] OR "chronicization"[All Fields] OR "chronics"[All Fields])) AND ("movement"[MeSH Terms] OR "movement"[All Fields] OR "movements"[All Fields] OR "movement s"[All Fields] OR ("motor"[All Fields] OR "motor s"[All Fields] OR "motoric"[All Fields] OR "motorically"[All Fields] OR "motorics"[All Fields] OR "motoring"[All Fields] OR "motorisation"[All Fields] OR "motorised"[All Fields] OR "motorization"[All Fields] OR "motorized"[All Fields] OR "motors"[All Fields]))

Translations

stroke: "stroke"[MeSH Terms] OR "stroke"[All Fields] OR "strokes"[All Fields] OR "stroke's"[All Fields]

rehabilitation: "rehabilitant"[All Fields] OR "rehabilitant's"[All Fields] OR "rehabilitants"[All Fields] OR "rehabilitate"[All Fields] OR "rehabilitated"[All Fields] OR "rehabilitates"[All Fields] OR "rehabilitating"[All Fields] OR "rehabilitation"[MeSH Terms] OR "rehabilitation"[All Fields] OR "rehabilitations"[All Fields] OR "rehabilitative"[All Fields] OR "rehabilitation"[Subheading] OR "rehabilitation's"[All Fields] OR "rehabilitational"[All Fields] OR "rehabilitator"[All Fields] OR "rehabilitators"[All Fields]

neurorehabilitation: "neurological rehabilitation"[MeSH Terms] OR ("neurological"[All Fields] AND "rehabilitation"[All Fields]) OR "neurological rehabilitation"[All Fields] OR "neurorehabilitation"[All Fields] OR "neurorehabilitative"[All Fields]

acute: "acute"[All Fields] OR "acutely"[All Fields] OR "acutes"[All Fields]

subacute: "subacute"[All Fields] OR "subacutely"[All Fields]

chronic: "chronic"[All Fields] OR "chronical"[All Fields] OR "chronically"[All Fields] OR "chronicities"[All Fields] OR "chronicity"[All Fields] OR "chronicization"[All Fields] OR "chronics"[All Fields]

movement: "movement"[MeSH Terms] OR "movement"[All Fields] OR "movements"[All Fields] OR "movement's"[All Fields]

motor: "motor"[All Fields] OR "motor's"[All Fields] OR "motoric"[All Fields] OR "motorically"[All Fields] OR "motorics"[All Fields] OR "motoring"[All Fields] OR "motorisation"[All Fields] OR "motorised"[All Fields] OR "motorization"[All Fields] OR "motorized"[All Fields] OR "motors"[All Fields]

1. *Search strategy for rehabilitation research in chronic stroke*

**Search: ((((stroke) AND (rehabilitation OR neurorehabilitation)) NOT(acute OR subacute)) AND (chronic)) AND (movement OR motor)**

((("stroke"[MeSH Terms] OR "stroke"[All Fields] OR "strokes"[All Fields] OR "stroke s"[All Fields]) AND ("rehabilitant"[All Fields] OR "rehabilitants"[All Fields] OR "rehabilitate"[All Fields] OR "rehabilitated"[All Fields] OR "rehabilitates"[All Fields] OR "rehabilitating"[All Fields] OR "rehabilitation"[MeSH Terms] OR "rehabilitation"[All Fields] OR "rehabilitations"[All Fields] OR "rehabilitative"[All Fields] OR "rehabilitation"[MeSH Subheading] OR "rehabilitation s"[All Fields] OR "rehabilitational"[All Fields] OR "rehabilitator"[All Fields] OR "rehabilitators"[All Fields] OR ("neurological rehabilitation"[MeSH Terms] OR ("neurological"[All Fields] AND "rehabilitation"[All Fields]) OR "neurological rehabilitation"[All Fields] OR "neurorehabilitation"[All Fields] OR "neurorehabilitative"[All Fields]))) NOT ("acute"[All Fields] OR "acutely"[All Fields] OR "acutes"[All Fields] OR ("subacute"[All Fields] OR "subacutely"[All Fields]))) AND ("chronic"[All Fields] OR "chronical"[All Fields] OR "chronically"[All Fields] OR "chronicities"[All Fields] OR "chronicity"[All Fields] OR "chronicization"[All Fields] OR "chronics"[All Fields]) AND ("movement"[MeSH Terms] OR "movement"[All Fields] OR "movements"[All Fields] OR "movement s"[All Fields] OR ("motor"[All Fields] OR "motor s"[All Fields] OR "motoric"[All Fields] OR "motorically"[All Fields] OR "motorics"[All Fields] OR "motoring"[All Fields] OR "motorisation"[All Fields] OR "motorised"[All Fields] OR "motorization"[All Fields] OR "motorized"[All Fields] OR "motors"[All Fields]))

Translations

stroke: "stroke"[MeSH Terms] OR "stroke"[All Fields] OR "strokes"[All Fields] OR "stroke's"[All Fields]

rehabilitation: "rehabilitant"[All Fields] OR "rehabilitant's"[All Fields] OR "rehabilitants"[All Fields] OR "rehabilitate"[All Fields] OR "rehabilitated"[All Fields] OR "rehabilitates"[All Fields] OR "rehabilitating"[All Fields] OR "rehabilitation"[MeSH Terms] OR "rehabilitation"[All Fields] OR "rehabilitations"[All Fields] OR "rehabilitative"[All Fields] OR "rehabilitation"[Subheading] OR "rehabilitation's"[All Fields] OR "rehabilitational"[All Fields] OR "rehabilitator"[All Fields] OR "rehabilitators"[All Fields]

neurorehabilitation: "neurological rehabilitation"[MeSH Terms] OR ("neurological"[All Fields] AND "rehabilitation"[All Fields]) OR "neurological rehabilitation"[All Fields] OR "neurorehabilitation"[All Fields] OR "neurorehabilitative"[All Fields]

acute: "acute"[All Fields] OR "acutely"[All Fields] OR "acutes"[All Fields]

subacute: "subacute"[All Fields] OR "subacutely"[All Fields]

chronic: "chronic"[All Fields] OR "chronical"[All Fields] OR "chronically"[All Fields] OR "chronicities"[All Fields] OR "chronicity"[All Fields] OR "chronicization"[All Fields] OR "chronics"[All Fields]

movement: "movement"[MeSH Terms] OR "movement"[All Fields] OR "movements"[All Fields] OR "movement's"[All Fields]

motor: "motor"[All Fields] OR "motor's"[All Fields] OR "motoric"[All Fields] OR "motorically"[All Fields] OR "motorics"[All Fields] OR "motoring"[All Fields] OR "motorisation"[All Fields] OR "motorised"[All Fields] OR "motorization"[All Fields] OR "motorized"[All Fields] OR "motors"[All Fields]
